# Supplementary material for: Complexome profiling of the Chlamydomonas psb28 mutant reveals TEF5 as an early PSII assembly factor
Source: Plant Cell. 2025 Mar 17;37(6):koaf055. doi: 10.1093/plcell/koaf055 (PMC12164590; doi:10.1093/plcell/koaf055)
Supplement: koaf055_Supplementary_Data [file koaf055_supplementary_data.zip › psb28_interactive.zip!/plots/intensity/qualityPlots/peptides/NIP1_Cre02.g119550.t1.2.html]

### Cre02.g119550.t1.2

Trivial name: NIP1  
SeqCoverage [median]: 55.20  
Mass: 9.95 kDa  
  
 

| Sequence | MissedCleavage | Mass [Da] | wtIntensity | muIntensity | Score |
| --- | --- | --- | --- | --- | --- |
| AAELSALK | 0 | 801.46 | 20249.33 | 27108.71 | 130.22 |
| AIEDLFFSK | 0 | 1068.55 | 5833.44 | 33838.92 | 147.19 |
| AIEDLFFSKEDQR | 1 | 1596.78 | 854.04 | 5222.16 | 105.86 |
| EQSDLADR | 0 | 932.42 | 407.02 | 1590.77 | 85.16 |
| EQSDLADRHAAEGAK | 1 | 1596.75 | 634.11 | 0.00 | 55.72 |
| VKEQSDLADR | 1 | 1159.58 | 21115.67 | 27300.65 | 122.69 |
| VKEQSDLADRHAAEGAK | 2 | 1823.91 | 19162.24 | 18972.17 | 139.90 |
| YDLPKDVLEK | 1 | 1218.65 | 790.00 | 10245.11 | 89.81 |
|  |  |  | 69045.8 | 124278.5 |  |
